# Up-Regulatory Effects of Curcumin on Large Conductance Ca^2+^-Activated K^+^ Channels

**DOI:** 10.1371/journal.pone.0144800

**Published:** 2015-12-16

**Authors:** Qijing Chen, Jie Tao, Hongya Hei, Fangping Li, Yunman Wang, Wen Peng, Xuemei Zhang

**Affiliations:** 1 Department of Pharmacology, School of Pharmacy, Fudan University, 826 Zhangheng Road, Pudong New District, Shanghai, 201203, China; 2 Department of Nephrology, Putuo Hospital, Shanghai University of Traditional Chinese Medicine,164 Lanxi road, Shanghai, 200062, China; University of Hull, UNITED KINGDOM

## Abstract

Large conductance Ca^2+^-activated potassium channels (BK) are targets for research that explores therapeutic means to various diseases, owing to the roles of the channels in mediating multiple physiological processes in various cells and tissues. We investigated the pharmacological effects of curcumin, a compound isolated from the herb *Curcuma longa*, on BK channels. As recorded by whole-cell patch-clamp, curcumin increased BK (α) and BK (α+β1) currents in transfected HEK293 cells as well as the current density of BK in A7r5 smooth muscle cells in a dose-dependent manner. By incubating with curcumin for 24 hours, the current density of exogenous BK (α) in HEK293 cells and the endogenous BK in A7r5 cells were both enhanced notably, though the steady-state activation of the channels did not shift significantly, except for BK (α+β1). Curcumin up-regulated the BK protein expression without changing its mRNA level in A7r5 cells. The surface expression and the half-life of BK channels were also increased by curcumin in HEK293 cells. These effects of curcumin were abolished by MG-132, a proteasome inhibitor. Curcumin also increased ERK 1/2 phosphorylation, while inhibiting ERK by U0126 attenuated the curcumin-induced up-regulation of BK protein expression. We also observed that the curcumin-induced relaxation in the isolated rat aortic rings was significantly attenuated by paxilline, a BK channel specific blocker. These results show that curcumin enhances the activity of the BK channels by interacting with BK directly as well as enhancing BK protein expression through inhibiting proteasomal degradation and activating ERK signaling pathway. The findings suggest that curcumin is a potential BK channel activator and provide novel insight into its complicated pharmacological effects and the underlying mechanisms.

## Introduction

Large conductance Ca^2+^-activated potassium (BK) channels, composed of α and tissue-specific β subunits, are present in a wide variety of cell types and are especially abundant in some of the excitable cells such as smooth muscle cells, neurons, and endocrine cells [1]. BK channels mediate many physiological processes including the maintaining of vasomotor tone, control of cell excitability and neurotransmitter release, cell proliferation, differentiation and apoptosis, etc. [2–4].

BK channels can be modulated by multiple factors. Besides membrane potential and intracellular Ca^2+^ concentration, other BK channel regulators include protein kinase A (PKA), protein kinase C (PKC), protein kinase G (PKG), steroid hormones, gases (NO, H_2_S) and drugs, etc [5, 6]. In light of the involvement of BK channels in various pathophysiological conditions, the regulations of their activity have been extensively studied, and BK openers are sought as novel avenues to the treatment of diseases such as stroke, epilepsy, asthma, cancer, and arterial ischemic heart disease [7]. Although many attempts have been made, only researches on four BK channel openers have been progressed to clinical development so far, yet all but Andolast^®^ have been discontinued [7].

Curcumin is a phenolic compound isolated from the herb *Curcuma longa*. Due to its effects on a diverse range of molecular targets [8], curcumin has been evaluated for its therapeutic potentials for various diseases, including inflammatory illnesses, cancer, Alzheimer’s disease and acquired immune deficiency syndrome (AIDS) [9–11]. Recent studies have shown that curcumin increases intracellular Ca^2+^ [12], inhibits voltage-dependent K^+^ channels [13] and activates the nociceptor ion channel TRPA1 [14], suggesting a role of curcumin in modulating ion channel activities. However, whether curcumin exerts any pharmacological effect on BK channel remains elusive. In this study, we have explored the regulatory effect of curcumin on the exogenous BK channels in HEK293 cells and the endogenous BK channels in A7r5 cells by electrophysiology and biochemical approaches. Here, we report that curcumin up-regulates BK channel via acute modulation possibly by its direct interaction with BK channel, and through chronic effect by the increase of BK protein level.

## Methods

### Cell culture and transfection

The functional characterization of curcumin (Sigma-Aldrich, USA, C7727, Purity ≥94%) was performed on the HEK293 cells overexpressing exogenous BK channels and A7r5 cells with endogenous BK, respectively. HEK293 and A7r5 cell lines were purchased from Shanghai cell bank of Chinese Academy of Science and cultured in Dulbecco’s modified Eagle medium (DMEM; Life Technologies, USA) supplemented with 10% fetal bovine serum (FBS; Hyclone, USA). Dishes for cell culture were incubated at 37°C in an incubator with a humidified atmosphere containing 5% CO_2_, and subcultured about every 2–3 days. HEK293 cells were co-transferred with 1 μg per well of pCMV-myc-BK or pIRES-hsloα-β1 plasmid (gifts from N.W. Davies, University of Leicester, UK and J.D. Lippiat, University of Leeds, UK) and 0.5 μg per well of pEGFP-N2 plasmid (Life Technologies, USA) using 0.75 μL per well of Lipofectamine 3000 and 1 μL per well of P3000 (Invitrogen, USA) for transient transfection experiments. The GFP fluorescence was used to identify the transfected cells. Cells were used for biochemical or electrophysiological studies 24–48 h after transfection.

### Whole-cell patch-clamp experiment

Whole cell recordings were carried out as described in detail previously [15–17]. Briefly, the bath solution for BK channels contained the following components (in mM): NaCl 135, KCl 5, MgCl_2_ 1.2, CdCl_2_ 2.5, HEPES 5, glucose 10 (pH 7.4 titrated with NaOH). Pipette solutions were composed of the following components (in mM): NaCl 10, KCl 117, MgSO_4_ 2, HEPES 10, MgATP 2, EGTA 1 (pH 7.2 titrated with KOH). The total Ca^2+^ to be added to give the desired free concentration was calculated using the program Maxchelator (http://www.stanford.edu/%7Ecpatton/maxc.html). Whole-cell voltage-clamp experiments were performed using an Axon Multiclamp 700B amplifier (Molecular Devices, USA) at room temperature. The recording electrodes were fabricated from glass capillary tubes by PC-10 (Narishige, Japan) as well as P97 (Sutter, USA) micropipette puller with the resistance of 2–4 MΩ and it was possible to compensate up to 85% of this without introducing oscillations into the current output of the patch clamp amplifier. Data acquisition and stimulation protocols were recorded and controlled by Clampex/Clampfit 10.2 software (Molecular Devices, USA). The holding potential was -80 mV for heterologous expressed BK in HEK293 cells and endogenous BK in A7r5 cell line. The recordings were done with the pulse of +100 mV for exogenous BK channels expressed in HEK293 and +120 mV for endogenous BK channels in A7r5 cells, respectively. For short-term dose-response experiments, curcumin (Sigma-Aldrich, USA, C7727, Purity≥94%) with each concentration were exerted to HEK293 and A7r5 cells within 15 min, respectively. For long-term dose-response experiments, A7r5 cells and HEK293 cells transfected with BK (α) were incubated in the medium containing 0, 1, 2, 5, 10 and 20 μM curcumin for 24 hours before the patch-clamp experiments, respectively.

### Intracellular free calcium detection

The level of intracellular free Ca^2+^ was measured by flow cytometry using a fluorescent dye Fluo-3 AM, which specifically bind to intracellular Ca^2+^. After exposed to 10 μM curcumin for 24 h, A7r5 cells were incubated in 5 μM Fluo-3 AM (Invitrogen, USA) for at 37°C in dark. Thirty minutes later, cells were harvested and washed twice with PBS. Mean fluorescence intensity (MFI) of 10,000 cells in each group was detected by flow cytometer at the excitation wavelength of 488 nm.

### Western blot analysis

HEK293 cells growing on culture dishes were washed twice with PBS, and then lysed in RIPA lysis buffer (50 mM NaCl, 1% Triton X-100, 0.1% SDS, 50 mM Tris-HCl pH 7.4, 1 mM EDTA, 1 mM phenylmethanesulfonyl fluoride) for 30 min at 4°C. The samples were then centrifuged (16000 × g for 15 min) to remove residual cell debris, and the supernatant was collected as total protein lysates. Protein concentrations were measured by BCA assay. Protein samples were mixed with 5× Laemmli buffer, incubated at 37°C for 20 min, separated on an 8% SDS-PAGE, and transferred to polyvinylidene difluoride (PVDF) membranes. The PVDF membranes were blocked in nonfat milk, incubated with primary antibody (anti-myc, 1:200, Santa Cruz, sc-40; anti-BK, 1:200, Alomone Labs, APC-021; anti-MAPK antibodies, 1:2000, Cell Signaling Technology, Phospho-MAPK Family Antibody Sampler Kit #9910 and MAPK Family Antibody Sampler Kit #9926) overnight at 4°C and with horseradish-peroxidase-conjugated secondary antibody for 2 h at room temperature. Immunoreactivity was detected with enhanced chemiluminescence (ECL; Pierce, USA) using Alpha Imager Detection System (AlphaInnotech, USA). The intensity of bands was quantified by ImageJ software (NIH, USA).

### Cell surface biotinylation

HEK293 cells were incubated with 1 mg/ml sulfo-NHS-SS-biotin (Pierce, USA) in Dulbecco’s phosphate buffered saline containing 1 mM CaCl_2_ and 1 mM MgCl_2_ (DPBS-Ca-Mg). After 40 min of biotinylation, the cells were washed twice with 100 mM glycine in DPBS—Ca-Mg to quench the reaction. After two additional washes with cold DPBS—Ca-Mg, the cells were scraped and solubilized with RIPA buffer same as used for protein isolation for western blot analysis. Lysates were incubated with immobilized NeutrAvidin beads (Pierce, USA) overnight at 4°C, and bound proteins were eluted with 50 μL 2×Laemmli sample buffer containing 200 mM DTT at 42°C for 1 h. After centrifugation at 14,000 g for 2 min, the eluents were collected for western blot analysis.

### Real-time RT-PCR

Total RNA was extracted from cells by using RNAiso Plus reagent (TaKaRa, China) and 0.5 μg of total RNA was used to synthesize the first-strand cDNA using the PrimeScript RT Master Mix (TaKaRa, China). Total of 2 μL of the first-strand cDNA solution was used in combination with the SYBR^®^ Premix Ex Taq^™^ solution for real-time RT-PCR. The primers used were: for BK (GenBank: NM_001014797.2), 5’-GCTGGATGACATCTGTGAAGG and 5’-GCACCAATGCTGAGAGCAAA; for GAPDH (GenBank: NM_001289746.1), 5’-ATGACCACAGTCCATGCCATC and 5’-CCTGCTTCACCACCTTCTTG. The amplicon size for BK and GAPDH is 103 bp and 271 bp, respectively. All experiments were run in triplicate. The real-time RT-PCR was run on iQ^™^5 real-time PCR detection system (Bio-Rad, CA, USA) with initial activation for 30 second at 95°C, followed by 40 cycles of 95°C for 5 seconds and 60°C for 30 seconds, and finally holding at 4°C. The threshold cycle (CT) of each target product was determined and normalized to the internal standard GAPDH. Difference in CT values (CT) of two genes was calculated using the ΔΔC_t_ method, ΔΔC_t_ = target group (CT of target genes—CT of GAPDH)—control group (CT of target genes—CT of GAPDH), and data were expressed as fold change to control.

### Cycloheximide chase experiment

At 24 h post transfection, the HEK293 cell culture medium was replaced by fresh DMEM plus 10% FBS with/without curcumin. Twelve hours later, cycloheximide (1 μg/ml) was added into the medium to stop protein synthesis. Cells were then harvested at 0, 3, 6, 9, 12, or 24 h after the addition of cycloheximide. Total cell lysates were analyzed by western blot analysis.

### Measurement of arterial isometric tension

Male Sprague-Dawley rats (250–300 g) were anesthetized with sodium pentobarbital (50 mg/kg i.p.) and the aortae were immediately excised, transferred to oxygenated (95% O_2_ /5% CO_2_) Krebs solution (in mM: 118 NaCl, 25 NaHCO_3_, 4.7 KCl, 1.2 KH_2_PO_4_, 1.2 MgSO_4_, 2.5 CaCl_2_, and 11 glucose) at 37°C, and then dissected into 3–4 mm rings. The presence of the endothelium was tested functionally by applying acetylcholine (Ach) (10 μM) on phenylephrine (PE) (1 μM) precontracted aortic rings and preparations demonstrating <70% relaxations in control group were discarded. The aortic rings were precontracted by 1 μM KCL and equilibrated until a stable resting tension was acquired. Curcumin of increasing concentrations (1, 5, 10, 20, 40 μM) were added cumulatively. In parallel experiments, a selective BK blocker, paxiline was added after the KCl-induced contraction, followed by administration of the same cumulative doses of curcucmin. The relaxation is expressed as a percentage of the steady-state tension (100%) obtained with 1 μM KCl.

### Ethics Statement

This study was carried out in strict accordance with the recommendations made in the Guidelines for the Care and Use of Laboratory Animals of the Fudan University School of Medicine. The protocol was approved by the Committee on the Ethics of Animal Experiments of the Fudan University of Shanghai (Permit Number: 2013030427). All surgery was performed under sodium pentobarbital anesthesia, and all efforts were made to minimize suffering.

### Data analysis

All statistical analysis was performed in the software Origin 8.5 (Northampton, Massachusetts, USA) and GraphPad PRISM 5 (GraphPad Software, Inc., USA). The degree of curcumin effect was calculated by expressing the remaining current after each drug exposure as a fraction of the current magnitude (or the current density magnitude) of the patch prior to the first drug exposure (i.e., fractional current remaining, I_f_). The current amplitude of endogenous BK channels in A7r5 cell line was calculated by the whole-cell current amplitude minus the remaining currents after exerting 10 μM paxilline. The current density (pA/pF) was calculated by the current amplitude of BK channels divided by the cell capacitance.

Fitting and calculation methods for electrophysiological data were described in details previously [15–17]. Dose-response curve for the enhancement percentage of curcumin on BK channel currents and currents density were drawn and fitted according to the Hill equation I = I_max_/(1+([curcumin]/EC_50_)^n^) (I_max_ is the maximum enhanced percentage of BK currents or currents density, [curcumin] represents the concentration of curcumin, EC_50_ is the half-maximal effective concentration and n denotes the curcumin concentration of half-maximal effect and the Hill coefficient, respectively.) The holding potentials were controlled at -80 mV, BK channel currents were evoked by the step pulses ranging from– 50 to +120 mV for the duration of 200 ms with 10 mV increments. For determining the voltage-dependent steady-state activation, the BK conductance was obtained by the formula: G = I/(V−Er) (I represents the BK channel currents (pA) at the command voltage V (mV), Er is the reversal potential of the BK channel). The conductance were normalized to the maximal value and the voltage dependent activation of the BK channel fitted by Boltzmann equation: f(x) = −1/(1+exp((x−V_1/2_)/k)) + 1. (V_1/2_ is the voltage when the half-maximal activation occurs, and k describes the slope of the fitted curve.)

Each parameter is presented as the mean ± SEM and was compared using Student’s paired t-test when there were only two groups involved and one-way ANOVA followed by Student-Newman-Keuls multiple comparisons tests in the remaining cases. *P* < 0.05 was considered significant statistically.

## Results

### Curcumin increases BK channel activity in HEK293 cells overexpressing BK (α)

**Fig 1:**
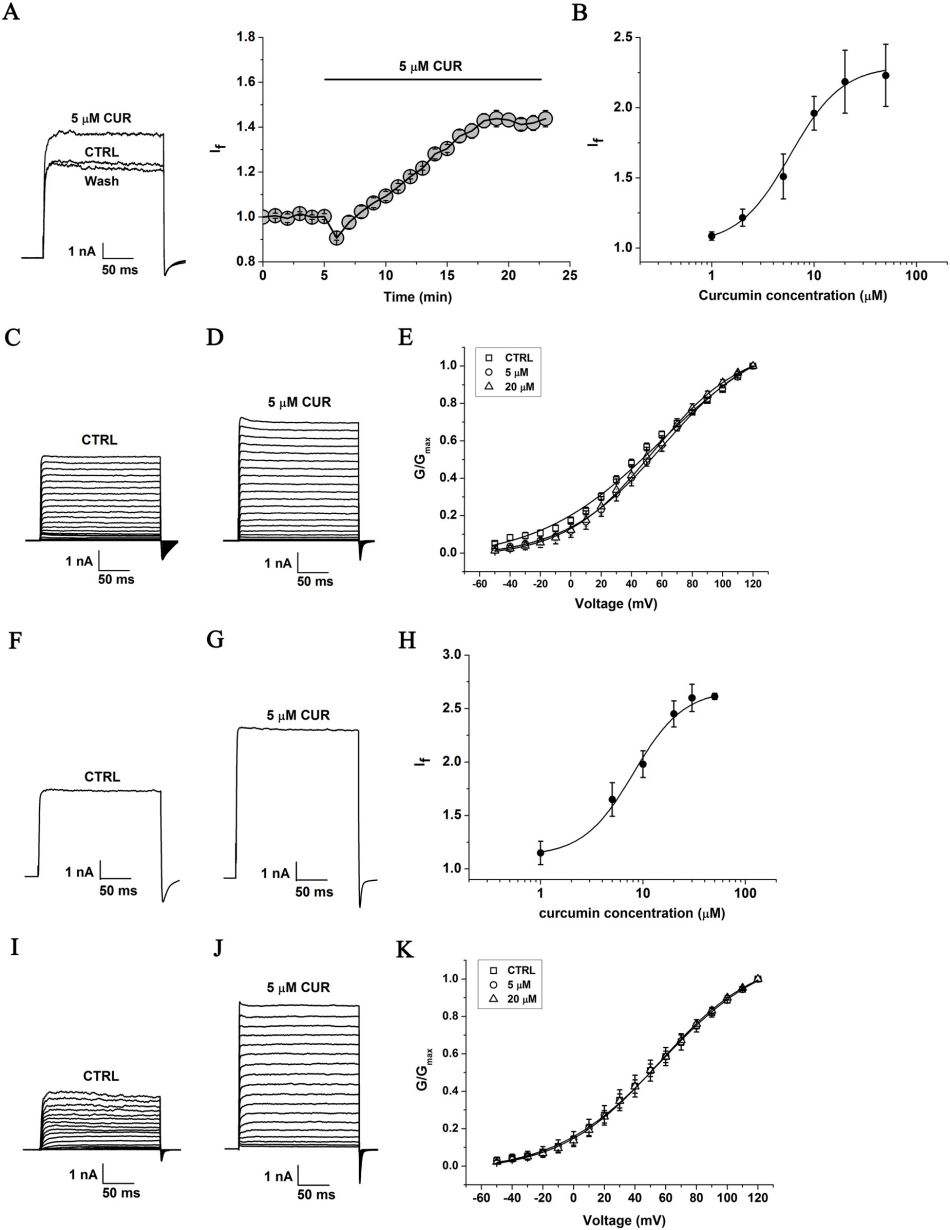
Effect of curcumin on exogenous BK (α) channel currents in HEK293 cells. (A) Representative whole cell current traces from HEK293 cells expressing BK channels before and after the perfusion of 5 μM curcumin (left) and the time course of the enhancement on BK (α) channels by of curcumin (right). With 3 μM free Ca^2+^ in the pipette solution, the holding voltage was controlled at -80 mV and BK currents were evoked by the pulse of +100 mV. (B) The dose-dependence curve of curcumin enhancing BK currents was fitted by the Hill equation (see “Data analysis”). The EC_50_ value is 5.83±0.76 μM with a Hill coefficient of n = 1.71±0.30 (n = 8). (C) HEK293 cells were held at -80 mV, and the duration of 200 ms voltage steps were applied from -50 to +120 mV at 10 mV increments. Representative trace before treatment with curcumin was shown. (D) Representative trace manifests the effect of curcumin at 5 μM. (E) The plots of the normalized conductance were fitted well with Boltzmann function (“see Data analysis”). The voltage dependence of steady state activation curve was not shifted significantly in the presence of curcumin (n = 8). □ represents the curve of BK channels before exposure to curcumin. ○ represents the curve of BK channels after exposure to 5 μM curcumin. Δ represents the curve of BK channels after exposure to 20 μM curcumin. Representative whole cell current traces from HEK293 cells expressing BK channels before (F) and after (G) the perfusion of curcumin 5 μM for 24 hours. (H) The dose-dependence curve of enhanced BK current density by curcumin was fitted by the Hill equation (see “Data analysis”). The EC_50_ value is 8.05±0.97 μM with a Hill co-efficient of n = 1.77±0.45 (n = 5). (I) Representative trace of BK currents and (J) the trace manifested the effect of curcumin at the concentration of 5 μM. (K) The plots of the normalized conductance were fitted well with Boltzmann function (“see Data analysis”). The voltage dependence of steady state activation curve was not shifted significantly in the presence of curcumin (n = 9). □ represents the curve of BK channels before exposure to curcumin. ○ represents the curve of BK channels after exposure to 5 μM curcumin. Δ represents the curve of BK channels after exposure to 20 μM curcumin.

To investigate the effect of curcumin on BK channel activity, outward potassium currents were measured in HEK293 cells transiently overexpressing BK channel. The current amplitude of BK (α) channels was increased by curcumin at 5 μM with the free Ca^2+^ concentration in the pipette solution maintained at 3 μM (Fig 1A, left). The time course for the enhancement of curcumin on BK (α) channel activity is shown at right in Fig 1A (n = 4). The puncta of BK current amplitude achieved maximum after ~13 min exerting with 5 μM curcumin. The EC_50_ of the curcumin effects on the BK (α) channel was calculated to be 5.83±0.76 μM with a Hill coefficient of n = 1.71±0.30 according to the dose-response curve fitted by the Hill function (n = 8) (Fig 1B). Furthermore, the BK currents were elicited by the step pulses ranging from –50 to +120 mV sustaining 200 ms with the increments of 10 mV. The effects of curcumin on the voltage dependent activation of the BK channel expressed in HEK293 cells were analyzed as described in Data analysis. Compared with the control group, the half-maximal voltage (V_1/2_) as well as the slope of the fitted curve (k) of the BK channels were not significantly shifted by treating with curcumin at either 5 μM or 20 μM (p>0.05, n = 8, Fig 1E and Table 1). To study the longer term curcumin effect, HEK293 cells transiently overexpressing the BK (α) channel were incubated in the medium containing 0, 1, 2, 5, 10, 20 and 50 μM curcumin for 24 hours before the patch-clamp experiments. The currents of the BK (α) channel were increased significantly by 5 μM curcumin (Fig 1F and 1G). The EC_50_ of curcumin on current density of the BK (α) channel was assessed to be 8.05±0.97 μM with a Hill coefficient of n = 1.77±0.45 (Fig 1H, n = 5). In addition, the activation curve of the BK (α) channel as well as the half-maximal voltage (V_1/2_) of activation were not significantly shifted with the application of 5 μM and 20 μM curcumin, respectively (p>0.05, n = 9, Fig 1K and Table 1).

**Table 1:** The voltage dependent activation of BK channels in the absence and presence of curcumin. Significant difference between the groups of control and curcumin at the level of 0.05 was tested by one-way ANOVA. V_0.5_ is the voltage for half-maximal activation, k is the voltage constant.

Curcumin concentration	V_0.5_, mV	k	n
**BK (α) (short term)**			
CTRL	57.21±1.37	42.63±2.07	8
5 μM curcumin	60.29±1.14	*32.38±1.47	8
20 μM curcumin	54.41±0.49	*29.39±0.61	8
**BK (α) (long term)**			
CTRL	57.05±1.60	31.55±3.07	9
5 μM curcumin	57.04±1.40	34.27±1.43	9
20 μM curcumin	55.78±1.16	32.41±1.20	9
**BK (α+β1)**			
CTRL	45.24±1.23	24.60±0.55	5
5 μM curcumin	*39.6±1.58	*27.20±0.80	5
20 μM curcumin	*40.06±1.10	*28.81±0.99	5

### Curcumin increases BK channel activity in HEK293 cells overexpressing BK (α+β1)

**Fig 2:**
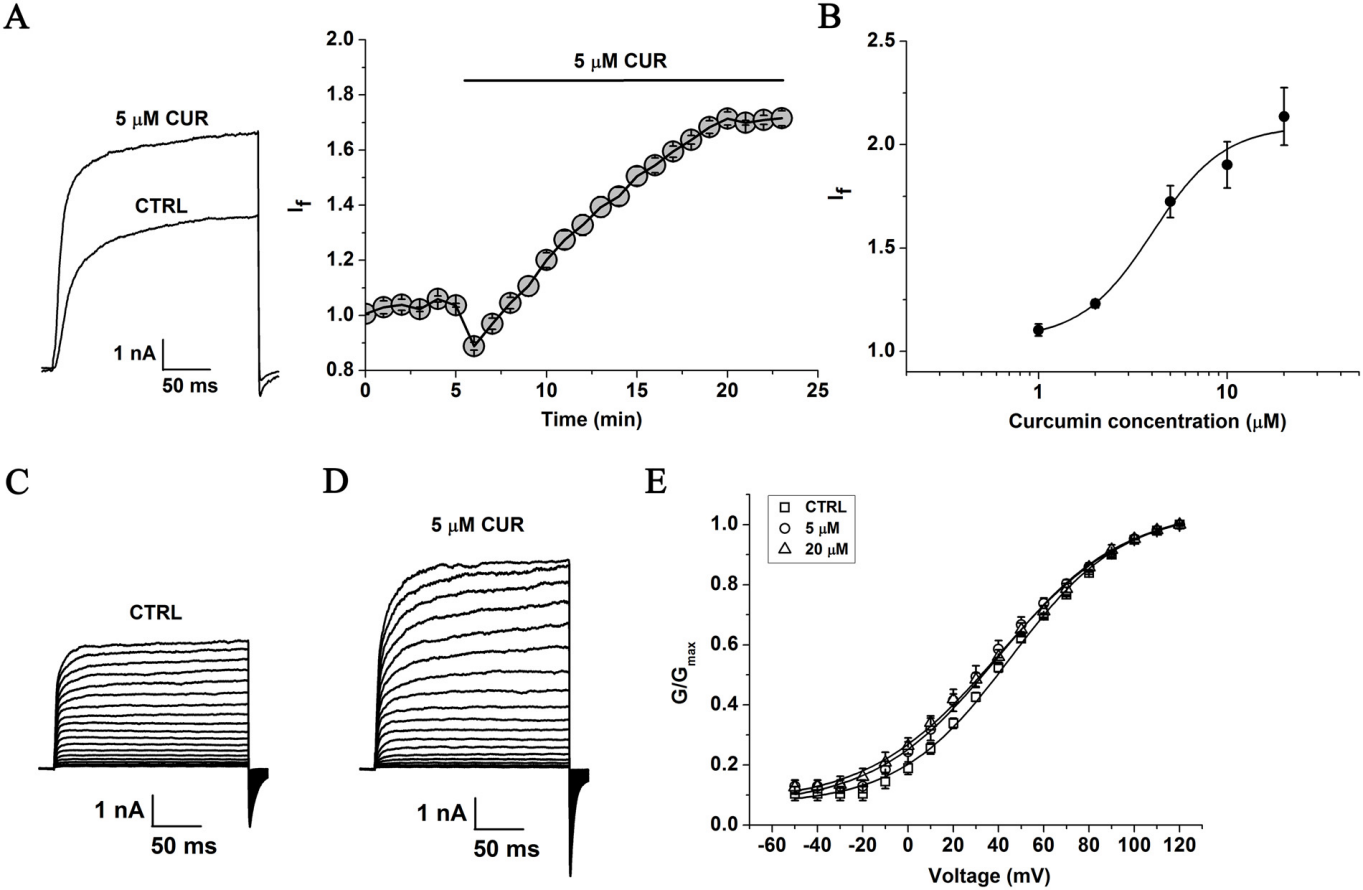
Effect of curcumin on exogenous BK (α+β1) channel currents in HEK293 cells. (A) Representative whole cell current traces from HEK293 cells expressing BK (α+β1) channels before and after the perfusion of 5 μM curcumin are presented at left and the time course for the enhancement on BK (α+β1) channels by curcumin at right. With 3 μM free Ca^2+^ in the pipette solution, the holding voltage was controlled at -80 mV and the BK currents were evoked by the pulse of +100 mV. (B) The dose-dependence curve of curcumin enhanced BK (α+β1) currents was fitted by the Hill equation (see “Data analysis”). The EC_50_ value is 4.02±0.67 μM with a Hill coefficient of n = 2.31±0.58 (n = 5). (C) HEK293 cells were held at -80 mV, and the duration of 200 ms voltage steps were applied from -50 to +120 mV at 10 mV increments. Representative trace before treatment with curcumin was shown. (D) Representative trace manifestes the effect of curcumin at the concentration of 5 μM. (E) The plots of the normalized conductance were fitted well with Boltzmann function (“see Data analysis”). The voltage dependence of steady state activation curve was not shifted significantly in the presence of curcumin (n = 5). □ represents the curve of BK (α+β1) channels before exposure to curcumin. ○ represents the curve of BK (α+β1) channels after exposure to 5 μM curcumin. Δ represents the curve of BK (α+β1) channels after exposure to 20 μM curcumin.

The BK (α+β1) channel currents were enhanced by 5 μM curcumin, with the free Ca^2+^ concentration in the pipette solution maintained at 3 μM (Fig 2A). The time course for the curcumin effect on the BK (α+β1) channels was carried out (Fig 2A, right, n = 4). The puncta of BK (α+β1) current amplitude achieved maximum after ~15 min exerting with 5 μM curcumin. The EC_50_ of curcumin on the BK (α+β1) channels was assessed to be 4.02±0.67 μM with a Hill coefficient of n = 2.31±0.58 (Fig 2B, 2C and 2D, n = 5). The BK (α+β1) currents were elicited by the step pulses ranging from –50 to +120 mV for 200 ms with 10 mV increments. The curcumin effects on the voltage dependence of steady-state activation were then analyzed as described in Data analysis. The perfusions with 5 μM and 20 μM curcumin resulted in the shifts of the activation curve of the BK (α+β1) channels as well as the half-maximal voltage (V_1/2_) of activation. V_1/2_ was changed to 39.6±1.58 at 5 μM curcumin and to 40.06±1.10 at 20 μM curcumin from 45.24±1.23 (control) (n = 5, P<0.05, Fig 2E and Table 1). The slope of the fitted curve (k) was changed to 27.16±0.82 at 5 μM curcumin and to 28.81±0.99 at 20 μM curcumin from 24.60±0.55 (control) (n = 5, P<0.05, Fig 2E and Table 1).

### Curcumin increases endogenous BK channel activity in A7r5 cells

**Fig 3:**
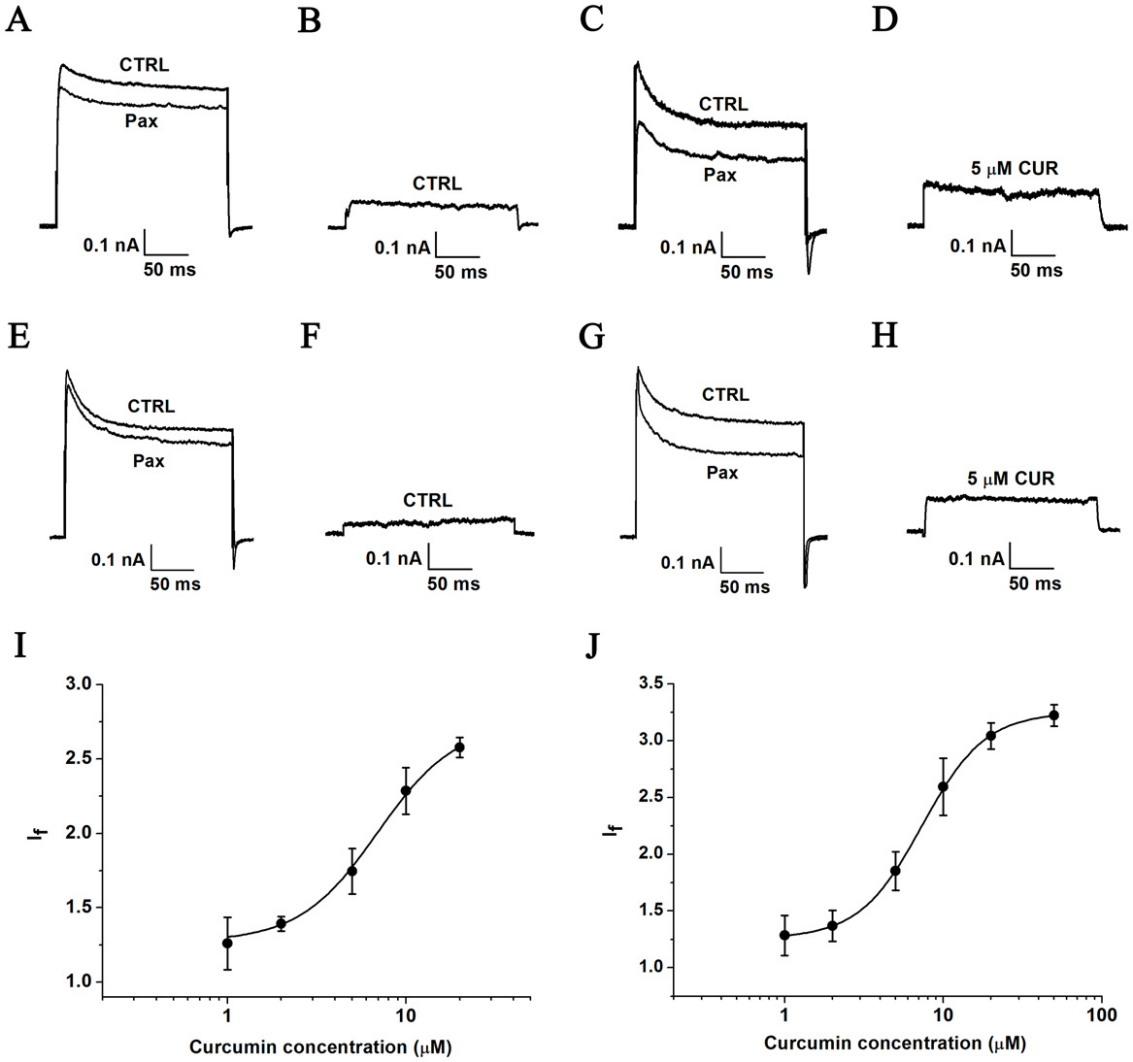
Effect of curcumin on the current density of endogenous BK channels in A7r5 cells. (A) Representative traces of whole cell currents from A7r5 cells in the absence of curcumin before and after application of 10 μM paxilline. The holding potential was -80 mV and the currents were evoked by +120 mV with 3 μM free Ca^2+^ in the pipette solution. (B) Representative traces of paxilline-sensitive currents in A7r5 cells. (C) The whole cell current traces of A7r5 cells in the presence of curcumin before and after application of 10 μM paxilline. (D) Representative traces of paxilline-sensitive currents in the presence of curcumin. (E) Representative traces of whole cell currents from A7r5 cells in the absence of curcumin before and after application of 10 μM paxilline. (F) Representative traces of paxilline-sensitive currents in A7r5 cells. (G) The whole cell current traces of A7r5 cells in the presence of curcumin for 24 hr before and after application of 10 μM paxilline. (H) Representative traces of paxilline-sensitive currents in the presence of curcumin for 24 hr. (I) The dose-dependence curve of curcumin enhancing current density of BK channels was fitted by the Hill equation (see “Data analysis”). The EC_50_ value was assessed to be 6.93±0.78 μM with a Hill coefficient of n = 2.00±0.55 (n = 6). (J) The dose-dependence curve of curcumin enhancing current density of BK channels was fitted by the Hill equation (see “Data analysis”). The EC_50_ value is 7.36±0.10 with Hill coefficient 2.19±0.05 (n = 6).

The amplitude of paxilline-sensive currents, considered equivalent to BK channel currents, were significantly increased in A7r5 cells, an excitatory smooth muscle cell line, after administering 5 μM curcumin, with the free Ca^2+^ concentration in the pipette solution kept at 3 μM, (Fig 3A–3D). The current density of paxilline-sensitive channels (BK channels) in A7r5 cells was increased by curcumin in a dose-dependent manner (Fig 3I). The EC_50_ of curcumin effect on the current density was assessed to be 6.93±0.78 μM with a Hill coefficient of n = 2.00±0.55 (n = 6). After a pre-exposure to 5 μM curcumin for 24 hours, an enhanced the current density of paxilline-sensitive currents was observed in A7r5 cells by patch-clamp recording (Fig 3E–3H). EC_50_ of curcumin on BK currents was 7.36±0.10 with Hill coefficient 2.19±0.05 (n = 6).

### Curcumin increases surface and total BK protein expressions without influencing BK gene transcription

**Fig 4:**
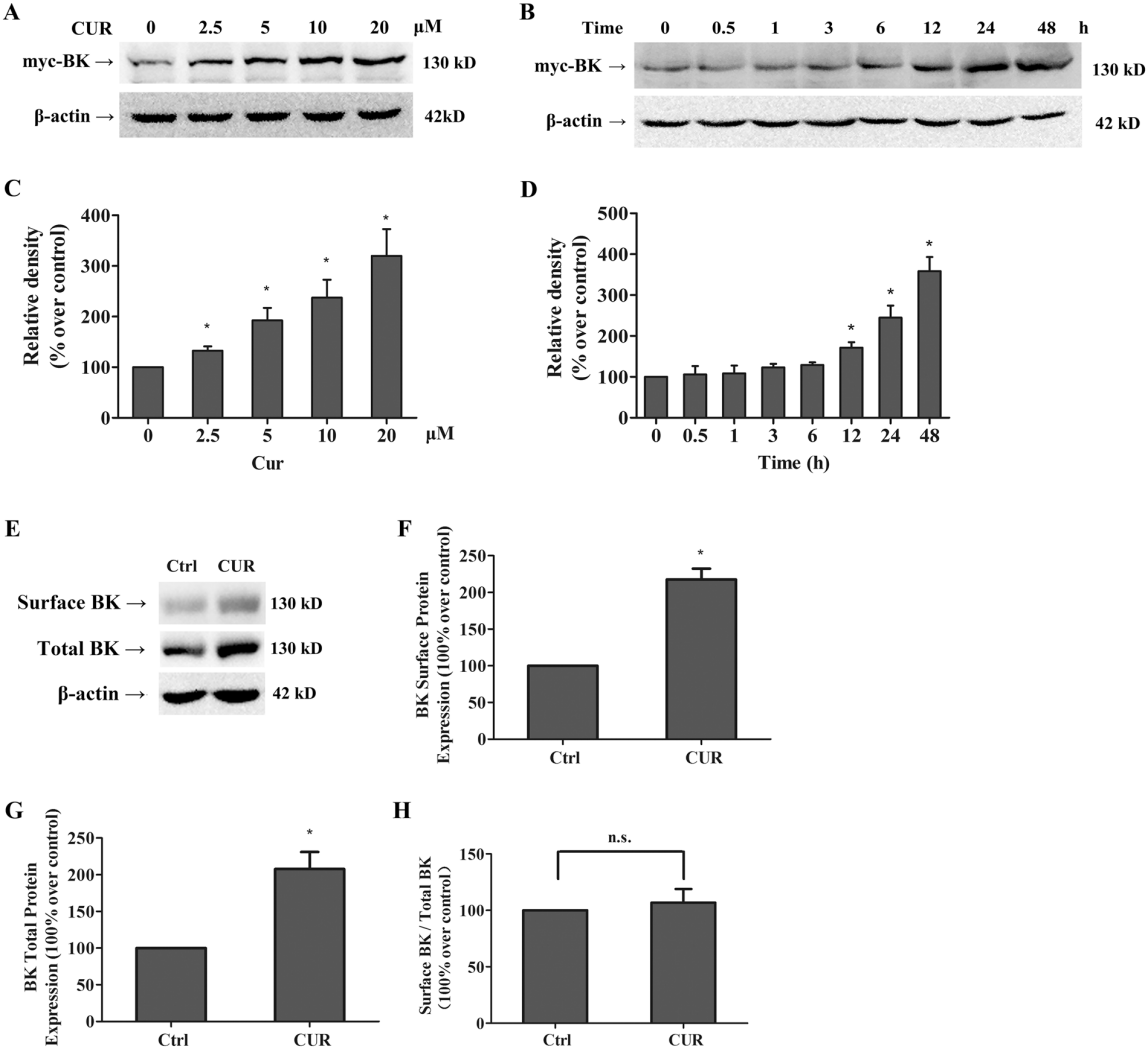
Curcumin increased the surface and total BK protein expressions in HEK293 cells. (A) Dose-dependent and time-dependent effects of curcumin on the BK protein in transfected HEK293 cells. Western blot analysis indicated that treatment with 2.5 to 20 μM curcumin for 24 h increased BK protein level in a concentration-dependent manner (n = 5). (B) The BK protein expression in HEK293 cells incubated with 10 μM curcumin for the time course of 0.5h to 48 h, showing an increase of BK protein level in response to curcumin (n = 3). Graphic representative of densitometric data of the total BK protein level shown in (C) and (D). (E) Representative western blot of total and surface BK protein expressions by cell surface biotinylation. The densitometric data showing the surface (F) and total (G) BK protein level and ratio of surface over total BK protein level (H) (n = 3). * *p* <0.05; n.s., not significant.

In a dose-dependent manner, curcumin treatment for 24 h increased BK protein levels in HEK293 cells transiently overexpressing BK channel (Fig 4A). At 10 μM, curcumin led to an increase in BK protein expression in the cells, starting at 12 h of treatment and continued steadily over a 48 h time period (Fig 4B).

To determine whether the increased BK currents by curcumin were the results of a higher abundance of BK channels in the plasma membrane, cell-surface biotinylation experiments were performed. As shown in Fig 4E, curcumin increased both the total and the surface BK protein expression by 2.07- and 2.17-fold respectively, compared to the control group. However, there was no significant difference in the ratio of surface to total BK protein expression between the curcumin treated group and the control group, suggesting that the surface BK protein expression is increased likely due to the increased total BK protein expression.

**Fig 5:**
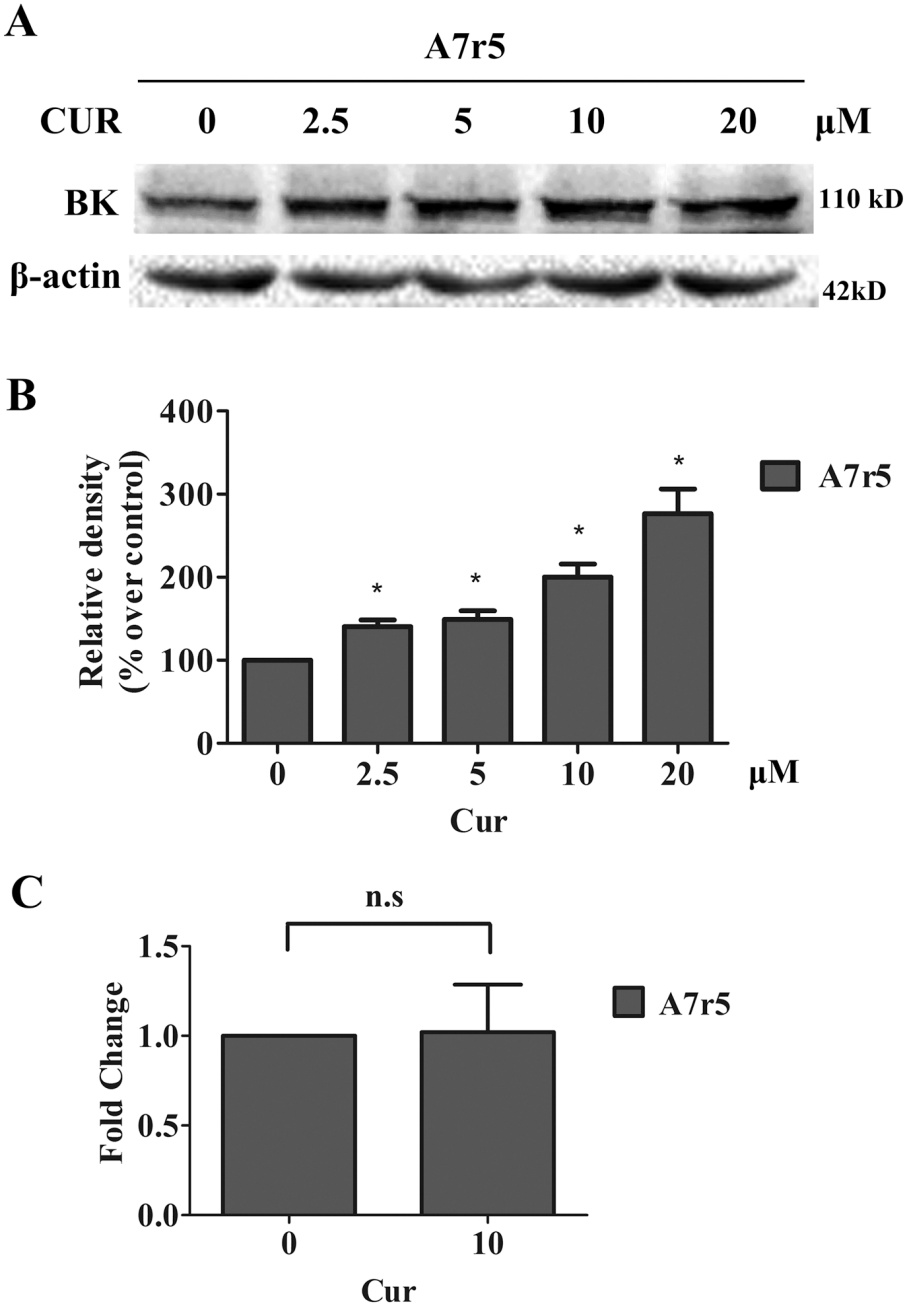
Effect of curcumin on BK protein abundance and BK mRNA in A7r5 cells. (A) Western blot analysis showed that treatment with curcumin at 0, 2.5, 5, 10, and 20 μM for 24h up-regulated the BK protein expression in A7r5 cells. * *p* <0.05 (n = 3). Graphic representative of densitometric data of the above total BK protein level are shown in (B). (C) Real-time RT-PCR for the measurement of BK mRNA levels with 10 μM curcumin treatment; the BK mRNA levels were normalized with GAPDH. The predicted amplicon size for BK and GAPDH is 103 bp and 271 bp, respectively. n.s., not significant (n = 3).

To determine whether curcumin also increases endogenous BK protein expression, western blot analysis was performed on A7r5 cell lysates treated with curcumin (0 to 20 μM) for 24 h. As shown in Fig 5A, curcumin increased endogenous BK protein expressions in a concentration-dependent manner.

This increase has occurred post gene transcription, as shown by real time PCR analysis on A7r5 cells, where the mRNA levels of BK channels were not significantly changed by 24h exposure of 10 μM curcumin, compared with the no treatment control (Fig 5C).

### Curcumin increases intracellular Ca^2+^ concentrations

**Fig 6:**
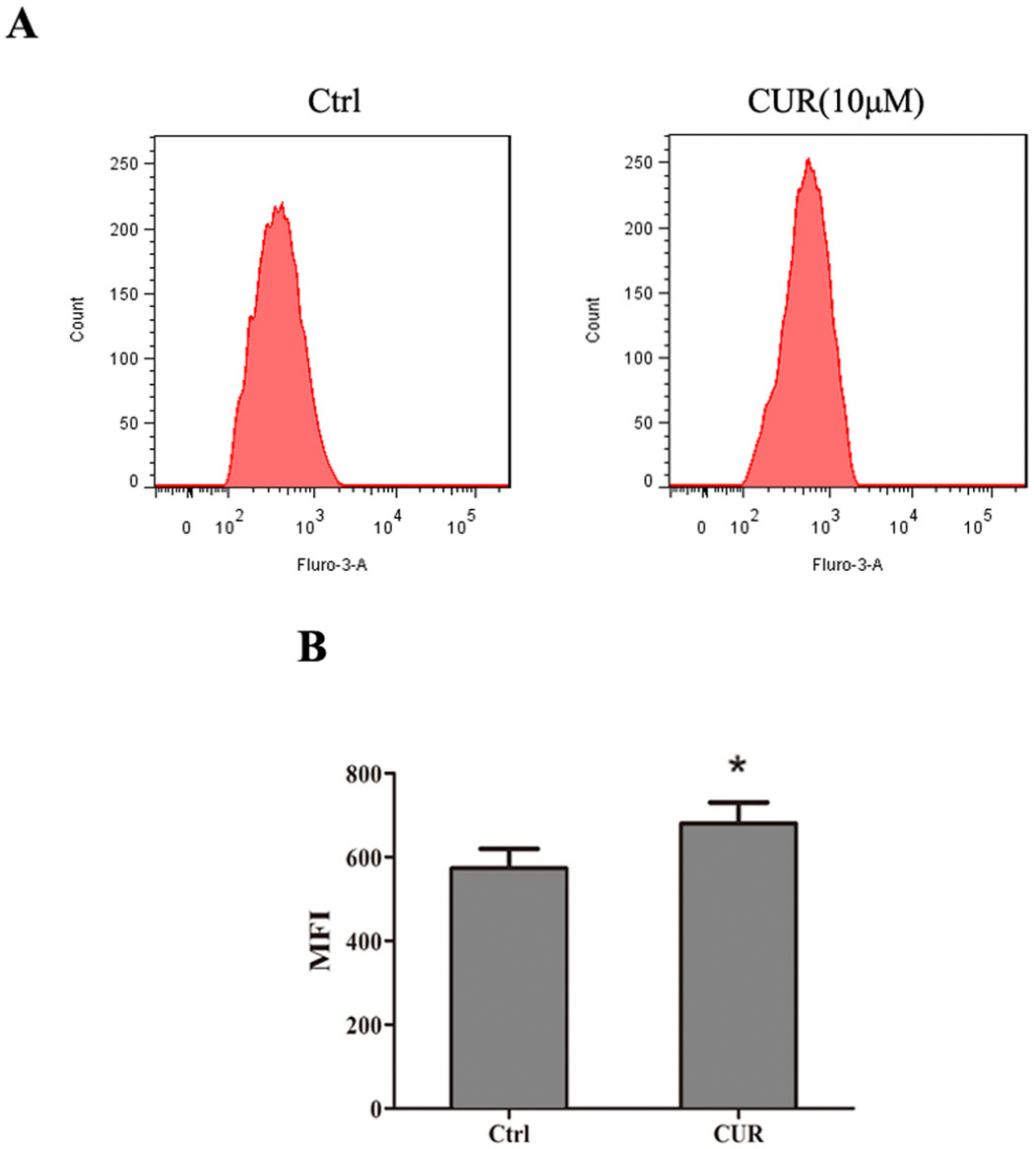
Effect of curcumin on intracellular free Ca^2+^ concentrations in HEK 293 cells. After treatment with 0 or 10 μM curcumin for 24 h, intracellular free Ca^2+^ were determined by flow-cytometric analysis stained with Fluo-3AM for 30 min. Results are expressed as mean fluorescent intensity.

We further investigated whether the activation of BK channel by curcumin is the result of the effects of curcumin on the intracellular Ca^2+^, as found in previous studies [12, 18]. Using flow cytometry with the fluorescent dye Fluo-3 AM, a slight increase of the intracellular free Ca2+ fluorescence was detected in HEK293 cells (Fig 6). The MFIs were 573 and 679, respectively.

### Curcumin increases BK protein stability by inhibiting BK degradations

**Fig 7:**
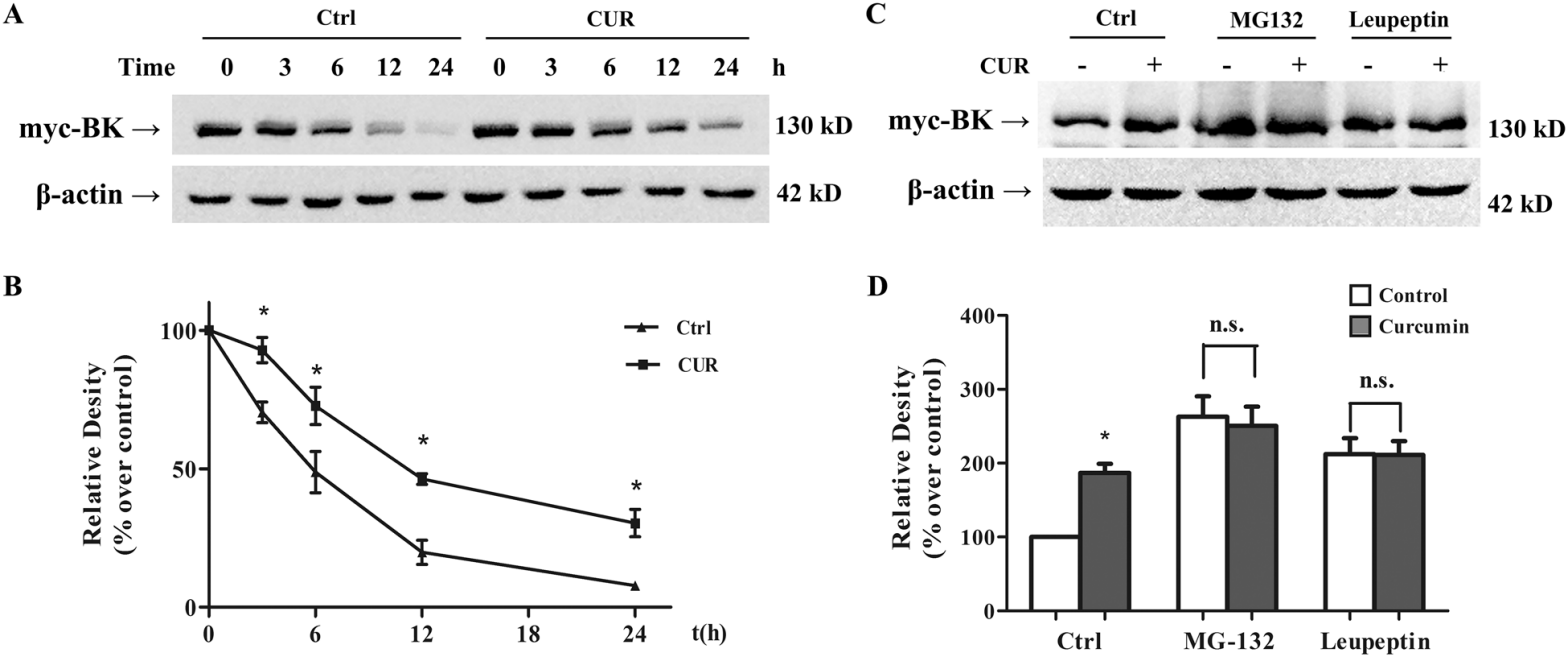
Curcumin increased BK protein stability via inhibition of degradation pathways. (A) Effect of curcumin on BK protein stability was examined with cycloheximide-chase assay. Western blot analysis showed that treatment with 10μM curcumin prolonged BK protein half-life. (B) Graphic representation of densitometric data showing the remaining BK protein level in Fig 7A (n = 3). (C) Effect of curcumin on BK protein in the presence and absence of proteasomal inhibitor, MG132. Western blot analysis showed that the increase of BK protein level was abolished by the proteasome inhibitor, MG132 (5 μM). Inhibitors were applied 1 h before the administration of curcumin. (D) Graphic representation of densitometric data of the BK protein level in Fig 7C (n = 3). * *p* < 0.05; n.s., not significant.

Since there was no indication that curcumin influences BK gene transcription, we investigated whether the elevation of BK protein level caused by curcumin is the result of enhanced protein stability. Cycloheximide-chase assays were performed to examine the degradation of BK channel in HEK293 cells. As shown in Fig 7, pretreatment with 10 μM curcumin resulted in a prolonged half-life of BK protein, suggesting that curcumin treatment stabilizes the BK channels by delayed their degradation.

We further examined whether the prolonged BK protein half-life by curcumin treatment is due to the reduction of BK degradation via ubiquitin/proteasome pathway. HEK293 cells were treated with 10 μM curcumin for 12 h with or without pretreatment of 5 μM MG-132, a proteasome inhibitor. As shown by western blotting, MG132 alone increased BK protein level by 2.6-fold (Fig 7C), indicating that the proteosome pathway is involved in BK degradation. Pretreatment with MG132 attenuated curcumin-induced increase of BK protein level, confirming that curcumin delayed BK protein degradation likely via inhibition of proteasomal pathway.

### Curcumin up-regulates BK protein expression via ERK1/2 signaling pathway

**Fig 8:**
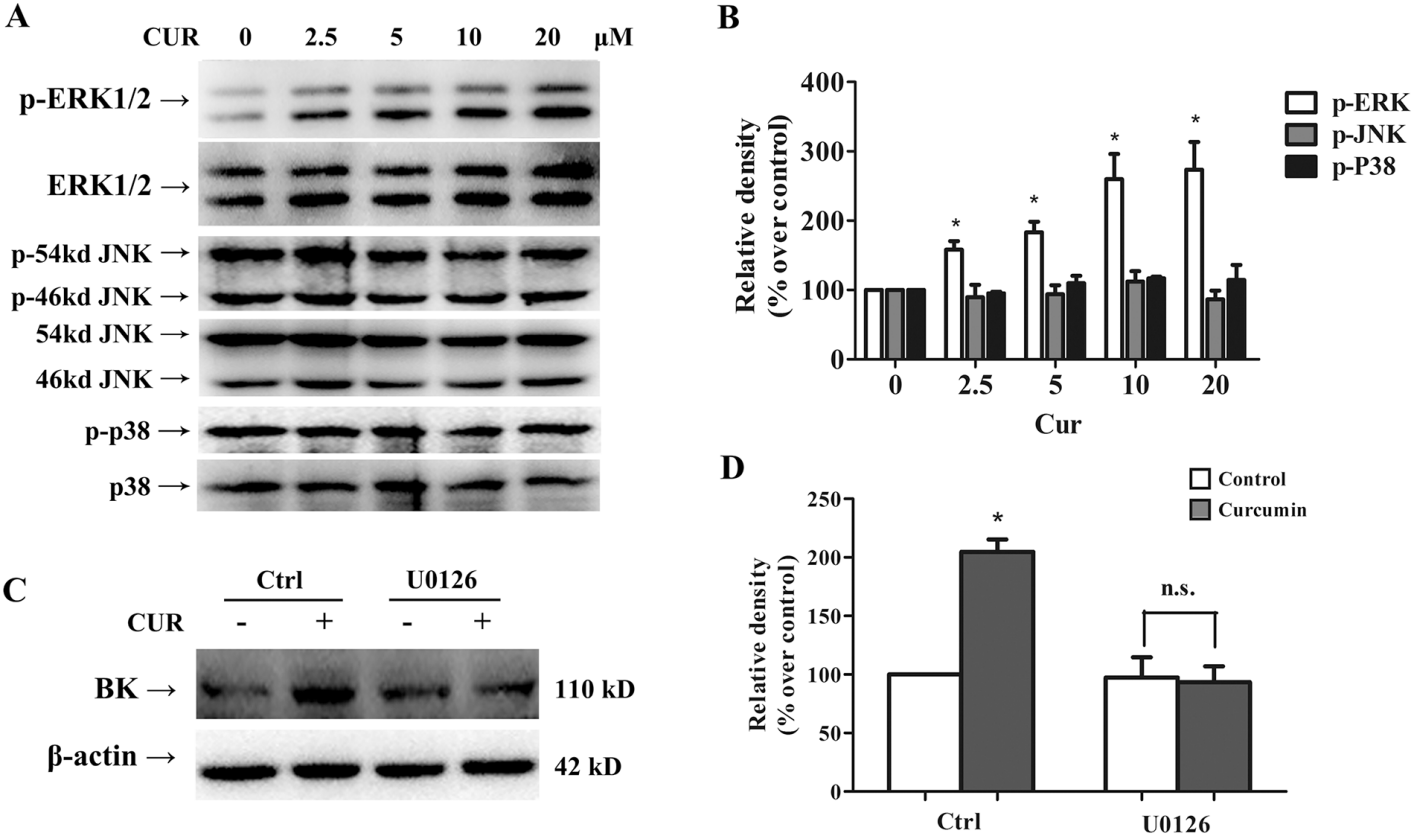
Curcumin up-regulated BK protein expression via ERK1/2 signaling pathway. (A) Effects of curcumin on ERK1/2, JNK, p38 phosphorylation. HEK293 cells were incubated with 10μM curcumin for 24 h, and the phosphorylation of ERK1/2, JNK, p38 were analyzed by Western Blot. (B) Graphic representation of densitometric data of the phosphorylation level of ERK1/2 (n = 3). (C) Western Blot analysis showed that the ERK1/2 inhibitors, U0126 (10 μM), abolished the effect of curcumin on BK protein. Inhibitors were applied 1 h before administration of curcumin. (D) Graphic representation of densitometric data show a protein level of BK (n = 4). * *p* < 0.05; ** *p* < 0.01; n.s., not significant.

The role of MAPK ERK1/2 signaling in the BK channel regulation has been well documented in several studies [19–21]. Here, we examined whether the effect of curcumin on BK protein expression is involved with MAPK ERK 1/2 signaling. As shown in Fig 8A, curcumin treatment significantly enhanced phosphorylation of ERK1/2 in a concentration-dependent manner in A7r5 cells, but it did not affect the phosphorylations of JNK and p38. Pre-treatment with U0126, a specific inhibitor of MAPK/ERK kinase (MEK) which is upstream of ERK in the MAPK ERK1/2 signaling cascade, abolished the curcumin-mediated up-regulation of BK protein in A7r5 cells (Fig 8C). Treatment with U0126 alone did not change BK protein expression in A7r5 cells.

### BK channel is involved in the vasorelaxant effect of curcumin

**Fig 9:**
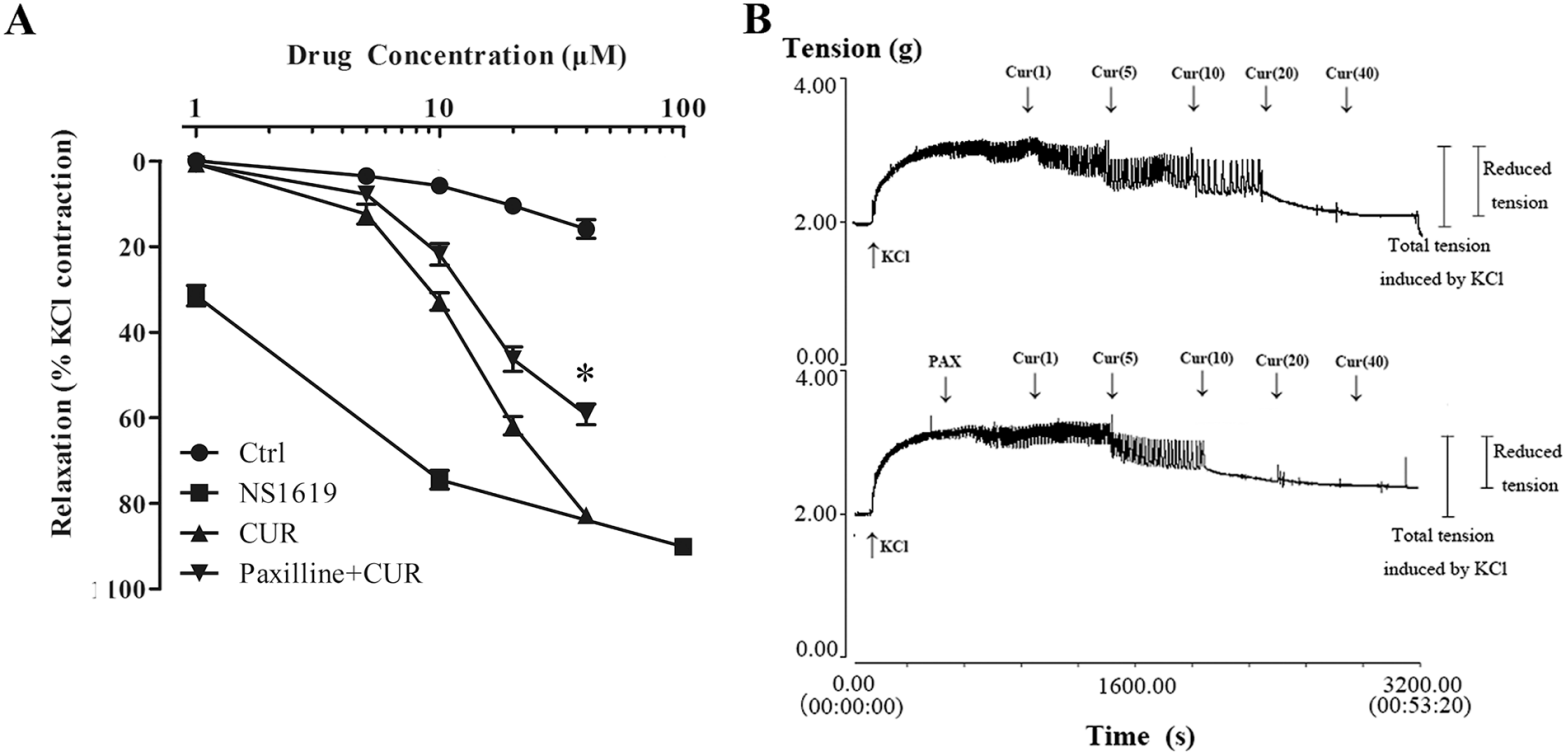
Effect of curcumin on the relaxation of the isolated rat aortic rings. (A) Concentration—response curves for the control, NS1619, curcumin (CUR) and paxalline (BK blocker) + CUR groups in the 20mM KCl-contracted arotic rings. Vasorelaxant effects are expressed as the percentage of the steady-state tension (100%) obtained with 20 mM KCl. Data points are presented as mean ± SE. (n = 4). *p < 0.05 compared to curcumin treatment alone. (B) Representative original traces of the vasorelaxant effects of curcumin, recorded on aortic rings precontracted with 20 mM KCl, in the absence or in the presence of paxilline. The curcumin was added cumulatively at the concentrations of 1, 5, 10, 20, 40 μM.

We tested curcumin for its ability to relax pre-contracted aorta rings. Curcumin showed significant vasorelaxant activities in a dose-dependent manner on 20 mM KCl-contracted rings (Fig 9). The relaxations induced by curcumin were 62.08% and 82.91%, at 20 and 40 μM curcumin applied, respectively. As reference, the BK channel opener, NS1619, caused almost full relaxations of 74.65% and 90.25%, with 10 and 100 μM NS1619 applied, respectively. The vasorelaxant effect of curcumin were significantly decreased in the presence of 1 μM paxilline, a selective BK blocker, in which the relaxations were reduced down to 46.61% and 59.47% (n = 4, P<0.05), respectively, when 20 and 40 μM curcumin were applied.

## Discussion

The ability of curcumin to regulate potassium channels has been recognized over the years. As previously described, curcumin inhibits Kv channels, including Kv1.3 and Kv11.1, and on the other hand, opens K_ATP_ channels [22]. The modulatory effect of curcumin is possibly due to the direct interaction with the channel protein, or its regulation of signal-transduction pathways [13, 23, 24]. In the present study, we demonstrated for the first time that curcumin up-regulates BK channel activity and protein expression level. We further delineated that curcumin stimulates BK protein expression by delaying BK protein degradation and by activating ERK1/2 signaling pathway. The results reveal that the BK channel is a relevant target for curcumin.

Our data suggests that the mechanisms for acute and long-standing regulatory effects of curcumin on BK channels might be different. The acute modulation is possibly caused by the direct interaction between curcumin and BK channels, whereas the chronic effect is possibly due to the increased BK protein abundance. We tested these effects of curcumin on the exogenous BK channel in transfected HEK293 cells and on the endogenous BK channel in A7r5 cells (Figs 1 and 3). The results indicated that, in both HEK293 cells and A7r5 cells, the chronic (24 hour) up-regulatory effect of curcumin was more potent than the acute regulation. In HEK293 cells, the current density was increased by 2.45±0.12 fold for 24 hour incubation and by 2.18±0.22 fold for acute perfusion, both with 20 μM curcumin. It was similarly observed in A7r5 cells, as the current density was enhanced by 3.04±0.12 fold for 24 hour incubation and by 2.58±0.07 fold for acute perfusion, respectively.

Our results also showed that curcumin increased the current amplitude of BK channels without altering the voltage-dependent activation, suggesting that curcumin neither acts on the voltage sensor of BK channels directly, nor induces the transient elevation of intracellular Ca^2+^ which would significantly shift the activation curve of BK channel in acute experiment. Previous studies have shown that curcumin increases intracellular free Ca^2+^ concentration in HepG2 cells [12]. Our data revealed that the intracellular Ca^2+^ in HEK293 cells was elevated by curcumin treatment for 24 hours (Fig 6). Taking these two results together, the intracellular Ca^2+^ accumulation is part of the chronic effects of curcumin, and possibly results in further kinase phosphorylation, such as ERK 1/2 signal pathway.

The β1-subunit of BK channel is expressed predominantly in arterial smooth muscle cells [25–27]. In our electrophysiological experiments, curcumin caused different effects on exogenous BK (α) and BK (α+β1) in HEK293 cells. The results showed that BK (α+β1) was more sensitive to curcumin than BK (α), with EC_50_ of 4.02±0.07 compared to 5.83±0.76 μM. The Hill coefficient of BK (α+β1) was 2.31±0.58, which indicated that curcumin would bind to two sites of BK (α+β1) channels (Fig 2B). Exposing to curcumin could shift the activation curve of BK (α+β1) channel by -5 mV (Table 1), suggesting that curcumin influences the gating of BK channels by acting on the β1 subunit.

Our study showed that curcumin increased the BK protein expression, not the BK mRNA level, suggesting that curcumin does not affect BK gene transcription, but rather stabilizes BK protein by reducing its degradation. As we shown by cycloheximide-chase experiment (Fig 7), pretreatment with curcumin slowed the BK degradation, confirming the notion that curcumin enhances the stability of BK protein. Previous studies including ours, have reported that the degradation of BK α-subunit involves the lysosomal pathway [28], and that the BK β1-subunit down-regulation is mediated by the ubiquitin-proteasome system (UPS) [29]. In the present study, we established that curcumin reduced the degradation of BK α-subunit as well, also through proteasomal pathway. Curcumin and its conjugates have been shown to inhibit UPS by inhibiting proteasome complex, COP9 signalosome and associated kinases [30]. The water soluble amino acid conjugates of curcumin were shown to exhibit antiproliferative effect by inhibiting proteasome human colon cancer and prostate cancer cells [31]. Curcumin was also found to reduce formation of infective viral particles from neuro2a cell line and protect neuronal cells from death through inhibiting of UPS [32]. These reports also support our notion that curcumin enhances BK protein level by delaying proteasomal degradation pathway mediated BK degradation.

MAPK family members can regulate BK channel activity, in opposite directions depending on cell types. Inhibition of P38 and ERK signaling were reported to stimulate BK channel activity in kidney principal cells and intercalated cells [19]. On the contrary, stimulating the MAPK pathway by insulin was shown to lead to BK channel activation both in hippocampal neurons and in human mesangial cells *in vitro* [20, 21]. Activation of p38 MAPK also resulted in opening of BK channels mediated by exchange protein activated by cAMP [33] in cerebellar neurons. Besides of cell type-specificity, these diverse effects of MAPK may be the result of direct phosphorylation of the BK channel protein or due to interaction with associated upstream or downstream proteins of BK channels. Our study showed that curcumin stimulated BK channel activity while increasing ERK 1/2 phosphorylation, but not JNK and p38 phosphorylation. Blockade of ERK1/2 with the specific inhibitor, U0126, abolished the curcumin-mediated up-regulation of BK protein levels. These results suggested that modulating ERK1/2 MAPK signaling pathway likely a part of the underlying mechanism for the up-regulation of BK protein level by curcumin.

As our results demonstrated that MAPK signaling and proteasomal degradation pathways are both involved in the curcumin enhanced BK activity and its protein expression, several studies have suggested that ERK1/2 regulates the proteasome-dependent protein degradation. The MAPK-ERK pathway activation was found to prevent the proteasome-dependent degradation of Fos-related antigen 1 (FRA-1) protein in colon carcinoma cells [34] and GATA3 protein in developing Th2 cells [35]. Stimulation of ERK1/2 signaling was also found to directly interacts with target proteins and then facilitate or prevent their degradation via ubiquitin-proteasome pathway [36, 37]. Activation of ERK1/2 could also indirectly protect proteins from proteasome-mediated degradation [38]. The precise relationship of ERK1/2 signaling and the curcumin-mediated delay of BK protein degradation needs to be further explored in our study, and ERK-dependent phosphorylation and ubiquitination of BK protein may play an important role in the process, as implied in these previous studies.

In addition, the effect of curcumin on BK channels was examined *ex vivo*. We found that curcumin possessed a significant vasorelaxant property in the vascular smooth muscles, which is consistent with the previous report that curcumin could induce relaxation of isolated porcine coronary arteries [39]. The relaxant effect of curcumin was attenuated in the presence of the BK channel blocker, paxilline, implying that this vasorelaxant effect is, at least in part, dependent on opening BK. Since BK channels are very abundant in smooth muscle cells [40], our findings suggest that curcumin plays an important role in regulating the BK-mediated vascular tone.

In conclusion, curcumin up-regulates BK channel activity by direct interaction as well as up-regulates BK protein level through delaying BK degradation via a proteasomal pathway and ERK1/2-mediated signaling mechanism. Our study demonstrated that curcumin is a potential BK channel up-regulator, providing a novel insight into the mechanism of multiple pharmacological activities of curcumin. Further study is needs to further delineate the underlying mechanisms and investigate the regulatory effect of curcumin on BK channel at tissue level and in *in vivo* study.
